# Validation of the death reflection scale among older people

**DOI:** 10.3389/fpsyg.2025.1541516

**Published:** 2025-02-11

**Authors:** Lukas Richter

**Affiliations:** ^1^Department of Social Sciences, St. Pölten University of Applied Sciences, Saint Pölten, Austria; ^2^Institute for Sociology and Social Research, Vienna University of Economics and Business, Vienna, Austria

**Keywords:** validation, death reflection scale, older people and aging, Austria, death awareness, cross-sectional online survey

## Abstract

**Background:**

Human beings possess the capacity to cognize their own mortality, which compels them to process death awareness. The present study seeks to validate the Death Reflection Scale (DRS) among older individuals, which measures growth-oriented cognitions and prosocial behavior following confrontation with death awareness.

**Materials and methods:**

DRS was validated using a cross-sectional online survey of older adults (50+). To assess configural, metric, and scalar measurement equivalence across age groups of older adults, a multi-group confirmatory factor analysis was conducted in conjunction with dynamic fit index cutoffs. Construct validity was evaluated using Pearson’s correlation and analysis of variance (ANOVA). The data were obtained from an online survey. The survey was conducted in May 2023 and people aged 50 and over were recruited from an online panel using quota sampling (by age, gender and federal state). A total of 1,806 individuals completed the survey.

**Results:**

Confirmatory factor analysis showed a good fit of the originally proposed 5-factor (CFI 0.949, SRMR 0.058, RMSEA 0.070) and bi-factor model (CFI 0.956, SRMR 0.067, RMSEA 0.067) with a general factor and five subscales. In light of several considerations, it is recommended that the DRS should be considered as a five-factor model, as originally proposed. Alpha ranges from 0.807 to 0.875 and Omega from 0.811 to 0.875, indicating good reliability. Partial scalar invariance was obtained, therefore mean comparisons can be made between groups of older people. Testing the construct validity showed only a partial confirmation. The exploratory analysis of the DRS with the Big Five personality traits revealed a correlation structure that can be plausibly explained by considering the facets of personality traits.

**Conclusion:**

The value of the DRS lies in its perspective that death awareness should not only be viewed as a threat, but rather as a potential for a positive and growth-oriented perspective on death awareness and has been validated for older adults. The present study demonstrated that mean comparisons could be conducted between groups of older adults. Tests of construct validity yielded inconclusive results, indicating the necessity for further analysis.

## Introduction

As much as our capacity for symbolic and abstract thought has contributed to our development into a highly technological society, it has also made us aware of our own mortality and thus of our own death. To avoid being overwhelmed or paralyzed by existential anxiety, people who are confronted with the fact of their mortality, according to the basic assumption of the Terror Management Theory, engage in behaviors that serve to increase their self-esteem and justify their cultural worldview (Greenberg et al., 1986, 1997; Pyszczynski et al., 2015). Over the past four decades, terror management research, which has been developing since the 1980s, has provided empirical support for the Terror Management Theory (TMT) and evidence that awareness of death has psychological and behavioral effects on individuals (Pyszczynski et al., 2010). The TMT postulates, among other things, that the pursuit of self-esteem and the processing of death awareness can be based on a wide range of antisocial − e.g., defending one’s worldview – as well as prosocial behaviors (Pyszczynski et al., 2004; Vail et al., 2012). Jonas et al. (2002) were able to demonstrate this in two experimental studies in the USA. The first study supported the prediction derived from TMT that people who are reminded of mortality have more positive attitudes toward charitable causes, while in the second study mortality salience increased the amount that people donated to charitable organizations, provided that the organizations supported an American cause. Other studies such as Zaleskiewicz et al. (2015) or Chen et al. (2020), as well as studies on life-threatening events such as earthquakes (Maki et al., 2019; Rao et al., 2011) or at the onset of the COVID-19 outbreak (Hu et al., 2020) also point to an increase in prosocial behavior.

Despite these findings, research on TMT has increasingly focused on the negative effects of death awareness (Vail et al., 2012), i.e., how existential fears can contribute to defensive distortions and aggressive protection of one’s cultural beliefs and self-esteem. Pyszczynski et al. (2000) themselves described the TMT as a “rather pessimistic theory about the role of core human fears in producing slavish conformity to cultural dictates and defensive conceptions of self” (p. 304). To address the positive or growth-oriented perspective, Cozzolino (2006) proposed a view of a “dual existential system” or, in other words, two sides of a death awareness coin (Cozzolino et al., 2004; Lykins et al., 2007), based on TMT and the self-determination theory (Deci and Ryan, 1980). Growth-oriented and defensive behaviors are considered to be the result of a variation (Cozzolino, 2006) in information processing (specific or abstract) and two competing motivational states (appetitive/approximative or aversive/avoidant). Grant and Wade-Benzoni (2009) built on these ideas, but considered the distinct forms of reaction on the basis of the hot/cool model of Metcalfe and Mischel (1999), which describes dynamics of self-regulation. What Cozzolino (2006) and Grant and Wade-Benzoni (2009) have in common is that they seek to distinguish positive aspects of death awareness from negative aspects and to provide a theoretical basis. The latter use the term death reflection to refer to those forms of death awareness that lead to prosocial motivational consequences. Based on these theoretical considerations that people can also cognitively process positive aspects of their own mortality, Yuan et al. (2019) define death reflection “as an individual’s deliberate cognitive processing of mortality that focuses on the positive aspects of death, which encompasses concrete behavioral intentions to realize such positive aspects” (p. 419) and developed the Death Reflection Scale (DRS) to measure death reflection. The DRS is a 15-item scale consisting of five subscales of three items each: (a) motivation to help (altruistic and prosocial behavior); (b) motivation to live (pursuit of life goals); (c) putting life in perspective (taking a more relaxed attitude); (d) leaving a personal legacy; (e) connecting with others. It was initially created and validated based on four studies with two smaller online samples, a sample of students and a sample of firefighters (Yuan et al., 2019). In addition to studies that already included the DRS in empirical models to account for death reflection without explicit validation (Ogbonnaya et al., 2024; Wei et al., 2021; Zampella and Benau, 2022), it was translated from English into German and the validity and reliability of the German version of the Death Reflection Scale, as well as measurement invariance across (younger) age and occupational groups, were tested in a sample of university members and health professionals in Germany during the COVID-19 pandemic (Ramsenthaler et al., 2022). Validation of the DRS in older age groups is therefore still needed, but seems worthwhile, as studies show that death awareness changes over the course of life (Bluntschli et al., 2015; Chopik, 2017; Maxfield et al., 2017). For example, results from Maxfield et al. (2014) support the hypothesis that younger and older adults differ in their reactions to death awareness, suggesting that older adults respond more strongly with a pro-social generative behavior.

The purpose of the present study is to (a) test the validity and reliability of the Death Reflection Scale, (b) determine its measurement invariance across older age groups, (c) conduct correlation analyses for chronological and perceived age to test construct validity, and (d) explore associations with Big Five personality traits using data from an online survey of the population aged 50 years and older.

To test the construct validity, hypotheses were formulated for each variable and dimension of the DRS:

*H1: Chronical age*: It can be hypothesized that motivation to help should increase (H1a) as altruistic behavior increases over lifespan (Sparrow et al., 2021) while a meta study shows that the will to live (H1b) and age are negatively associated (Bornet et al., 2021). A positive correlation (H1c) is expected between age and putting life into perspective, as an age-related change in motivation from extrinsic-instrumental to intrinsic-valuerational reduces the likelihood of perceiving problems as stressors (Aldwin et al., 2021) and contributes to maintaining emotional balance. Yuan et al. (2019) argue that in the context of the stages of psychosocial development toward generativity (Erikson and Erikson, 1998) and according to the socioemotional selectivity theory (Carstensen et al., 1999), people tend to emphasize making meaningful connections and leaving their legacy as they age. So it can be hypothesized that age will be positively associated with the DRS dimensions legacy (H1d) and connection to others (H1e).

*H2: Preceived age*: Subjective age predicts survival, correlates with molecular markers of aging (Voegeli et al., 2021) and is positively associated with life satisfaction, having a sense of meaning in life, optimism and successful aging (Ambrosi-Randić et al., 2018) while Westerhof and Wurm (2015) state in their theoretical model that negative self-perceptions of aging diminish psychological resources such as subjective well-being, control beliefs or will to live. In addition, studies show that subjective age is linked with loneliness (Bergman et al., 2024) and social activities (Montepare, 2020). Therefore, it can be hypothesized that perceived age will be negatively associated with the motivation to help others (H1a), motivation to live (H2b), putting life into perspective (H2c) and connections to others (H2e). Conversely, the perception of age and legacy (H2d) should be found to be positively correlated. This is based on the assumption that a negative evaluation of the self, if one considers oneself to be older than one’s chronological age, could encourage a reflection on death and thus also an increased reflection on one’s own legacy.

## Materials and methods

The following sections discuss the DRS, the additional variables used to test validity, the statistical methods and survey.

### Death reflection scale

As mentioned above, the Death Reflection Scale was developed by Yuan et al. (2019). It is a 15-item scale consisting of five subscales of three items each. Items are rated on a six-point scale from 1 (strongly disagree) to 6 (strongly agree). The range of additive subscale scores is 0–15 and the total score is 0–75. The German version was translated and proofed by Ramsenthaler et al. (2022). Yuan et al. (2019) tested several factor analytic models, with the 5-factor model showing the best fit: *χ*2 = 192.13, df = 80, CFI = 0.96, SRMR = 0.05; Cronbach’s alpha for the five factors ranged between 0.73 and 0.87. The results of the confirmatory factor analysis confirmed the considerations of Yuan et al. (2019). In contrast Ramsenthaler et al. (2022) demonstrated the best fit with a bi-factor model, followed by the 5-factor model: *χ*2 = 598.2, df = 8 0, CFI = 0.926, SRMR = 0.039; Cronbach’s alpha for the five factors ranges between 0.84 and 0.90 (see Table 1). Following these studies, the original conception as a 5-factor model in the context of older people will be tested in depth. However, the results of Ramsenthaler et al. (2022) are also taken into account and further factor analytic basic models are examined.

**Table 1:** Comparison of factor analytical baseline models of DRS (total sample).

	X^2^ (df)^1^	CFI	RMSEA [90% CI]	SRMR	AIC	BIC
Unidimensional model	7706.8 (90)*	0.452	0.217 [0.212, 0.221]	0.151	7766.78	7931.74
DFI cutoff values (uni)^3^	-	> 0.961	< 0.04	< 0.031	-	-
DFI cutoff values (uni)^4^	-	> 0.906	< 0.064	-	-	-
5-factor model	785.6 (80)*	0.949	0.070 [0.065, 0.074]	0.058	865.55	1085.50
Yuan et al. (2019)^2^	192.13 (80)*	0.96	-	0.039	-	-
Ramsenthaler et al. (2022)^2^	598.2 (80)*	0.926	0.061 [0.057, 0.066]	0.039	-	-
DFI cutoff values (5-factor)^3^	-	> 0.929	< 0.089	< 0.067	-	-
DFI cutoff values (5-factor)^4^	-	> 0.933	< 0.081	-	-	-
Second-order model	1024.2 (85)*	0.932	0.078 [0.074, 0.083]	0.083	1094.18	1286.64
DFI cutoff values (2nd order)^4^	-	> 0.930	< 0.080	-	-	-
Bi-factor model	689.2 (75)*	0.956	0.067 [0.063, 0.072]	0.067	779.19	1026.63
DFI cutoff values (bi-factor)^4^	-	> 0.95	< 0.081	-	-	-

^1^*, test statistically significant; ^2^Fit of the 5-factor model. Depending on sources some information is missing. ^3^OP method equivalent to Hu and Bentler (1999) calculated with Wolf and McNeish (2024). ^4^DDM method.

df, degrees of freedom; CFI, comparative fit index; RMSEA, Root mean square error of approximation; CI, confidence interval; SRMR, standardized root mean square residual; AIC, Akaike’s information criterion; BIC, Bayesian (Schwartz) information criterion.

### Additional variables

#### Age

Due to the target population of the study, people aged 50 and over at the time of the survey were included in the sample. The aim was to reflect the age structure of the older Austrian population. The sample included people aged between 50 and 93. In order to carry out tests for measurement invariance, three age groups of similar sample size were formed: 50–59 years (*n* = 670), 60–69 years (*n* = 524) and 70+ years (*n* = 612).

#### Perceived age

In addition to chronological age, subjective age was measured to compare the two values in order to identify discrepancies (Alonso Debreczeni and Bailey, 2021). Based on this comparison, three different age groups were constructed: those who felt younger (1), those who felt as old as their actual chronological age (2), and those who felt older (3).

#### Big Five

The short version of the Big Five Inventory (BFI-10) was used to measure personality structure. This instrument is considered a pragmatic alternative (Rammstedt et al., 2017) to the BFI-44 (John et al., 1991) for surveys with time constraints; see Rammstedt and John (2007) and Rammstedt et al. (2017) for detailed information on reliability. The scale has two items per personality dimension, rated on a five-point scale from not at all true (1) to completely true (5). The characteristics – extraversion, agreeableness, conscientiousness, neuroticism, openness – can take values between 1 (very low) and 8 (very high).

### Statistical analysis

The factor structure and comparison of factor analytical baseline models of DRS were examined by confirmatory factor analysis (CFA). The assessment of model fit is a central component of the evaluation of confirmatory factor analysis. In this context, the fixed cutoffs proposed by Hu and Bentler (1999) have gained considerable popularity. Nevertheless, methodological studies have indicated that cutoff values vary depending on the data and model characteristics, including the number of items or factors (Shi et al., 2019), degrees of freedom (Kenny et al., 2015) or the magnitude of the standardized loadings (McNeish et al., 2018). To address this issue, McNeish and Wolf (2023) propose a simulation-based method, which they refer to as dynamic fit index cutoffs. This method adapts the cutoffs to the specific model and data characteristics that are being evaluated. For the unidimensional and 5-factor model of DRS Omitted Paths (OP; estimator: maximum likelihood; cutoff precision: 3-decimal point; misspecification level: 1) was conducted as misspecification method (Wolf and McNeish, 2024). This method was chosen, because it mirrors Hu and Bentler (1999), to which Yuan et al. (2019) and Ramsenthaler et al. (2022) refer. All cutoff values are given in Table 1. For the 5-factor model, for example, the following values are obtained: Comparative Fit Index (CFI, > 0.929), Standardized Root Mean Square Residual (SRMR, < 0.089) and Root Mean Square Error Approximation (RMSEA, < 0.067). By contrast, Hu and Bentler (1999) recommend CFI > 0.95, SRMS <0.08 and RMSEA <0.06. For the two more complex baseline models, Wolf and McNeish (2024) strongly recommend an estimation using Direct Discrepancy Matrix (DDM) as misspecification method. No SRMR can be calculated, but the misspecification levels are standardized (based on the mean absolute discrepancy), which allows comparisons of cutoffs (CFI and RMSEA) between different models. These are calculated (estimator: maximum likelihood; cutoff precision: 3-decimal point; misspecification level: fair = mean absolute discrepancy 0.05) for all baseline models. Additionally, all models were compared via Akaike’s Information Criterion (AIC) and the Bayesian Information Criterion (BIC); smaller values indicate better fit. The following models were tested: (a) *unidimensional model* (a single factor explains the variance of all observed variables), (b) *correlated 5-factor model* (observed variables are grouped and act as indicators for latent factors), (c) *second-order model* (a higher factor causes each of the five first-order factors), (d) *bi-factor model* (a general factor directly affects the indicators and is orthogonal to the five specific factors). In addition, cutoff recommendations of Hair et al. (2014) were used to assess factor loadings and those of Cheung et al. (2024) for Alpha and Omega. All cases (*n* = 1,806) were included for the CFA as there were no missing data due to the survey design. However, the data are not normally distributed - see also Ramsenthaler et al. (2022). Therefore, the Bollen-Stine bootstrap procedure was applied (Bollen and Stine, 1992), which showed an acceptable fit.

Multi-group confirmatory factory analysis was carried out to test measurement invariance across three age groups (50–59; 60–69, 70+). An additional age group of 80+ would have been desirable. However, the sample of this group is too small (*n* = 121) to fulfill statistical requirements for CFA (Kyriazos, 2018). Four multi-group CFA models with increasingly restrictive assumptions on measurement equivalence were specified to test for measurement invariance (Putnick and Bornstein, 2016): (a) The *Configural invariance model* assumes the same factor structure across groups; model parameters are freely estimated. Invariance at this level means that the structure of the constructs is supported in the three age groups. (b) The *Metric invariance model* constrains the factor loadings to equality. Metric invariance means that the items contribute to the latent factor to a similar degree across age groups. (c) The *Scalar invariance model* further constrains the item intercepts to equality. If scalar invariance is given, this means that mean differences in the latent construct capture all mean differences in the common variance of the items and factor means can be compared across the age groups. The present analysis shows, however, that at least one item intercept differed between the age groups. Following Putnick and Bornstein (2016), the source of non-invariance was investigated by sequentially releasing item intercept constraints and retesting until a model of partial scalar invariance was achieved. (d) The *Strict invariance model* additionally constrains the item residuals to equality. As Putnick and Bornstein (2016) or Brown (2015) point out, it is an overly restrictive test and “prediction of a group equivalent observed score by the latent variable model does not rely on the condition of equal indicator error variances” (Brown, 2015, S. 262). Notwithstanding the fact that the model is frequently omitted, it will be included in this study in order to ensure completeness.

The total score and the scores of the 5-factor solution of the DRS for the given sample of older people are reported in the next step. In addition to skewness (0 = symmetrical distribution; cutoff: excellent −1/+1; acceptable −2/+2) and (‘excess’) kurtosis (0 = normal distribution; cutoff: excellent −1/+1; acceptable −2/+2 – George and Mallery, 2021), Cronbach’s Alpha (*α*) and, due to the criticism of *α* (Cho and Kim, 2015), McDonlad’s Omega (*ω*) was also calculated by Hayes and Coutts (2020) OMEGA macro. Pearson’s correlation and analysis of variance (ANOVA) were used to assess construct validity. Unless otherwise stated, the analysis was conducted using SPSS 28 and AMOS 28.

### Sample

The data were obtained from an anonymous cross-sectional online survey that examined death reflection, altruism and actual donation behavior of older people. The items regarding death reflection were introduced in the middle of the questionnaire. The entire questionnaire was pre-tested on the basis of 23 respondents (thinking aloud). The survey was conducted in May 2023 and people aged 50 and over were recruited from an online panel (panel size: approx. 55,000) using quota sampling (by age, gender and federal state) to ensure that the sample reflected the distribution of older people (50+) in Austria. Prior to participation, all individuals were informed of the study’s purpose, data protection, and their rights (see also section ethics statements). The survey was only initiated once informed consent had been obtained in accordance with ethical standards. To ensure sufficient power for model testing – for multi-group CFA, the size of each group needs to be considered (Jobst et al., 2023) – the minimum pre-specified sample size was 500 per age group (Meade and Lautenschlager, 2004).

## Results

A total of 1,806 individuals participated in the survey. As intended based on the quota corresponding to the distribution in the Austrian population (50+), the sample contains “”a slightly higher proportion of females (53.1%) and reflects the age structure (*M* = 64.6; SD = 9.6; categorized: 50–54 = 18.3%, 55–59 = 18.8%, 60–64 = 16.2%, 65–69 = 12.8%, 70–74 = 11.1% and 75+ = 22.8%). Further sample characteristics are: ISCED (0–2 = 6.3%, 3–4 = 79.4%, 5–6 = 14.3%), marital status (single = 12.5%, married = 59.8%, widowed = 6.8%, divorced = 20.9%), number of living children (0 = 21.5%, 1 = 24.6%, 2 = 35.4% 3+ = 18.5%) and household size (1 = 30.5%, 2 = 53.8%, 3+ = 15.7%).

Table 1 shows the results of the four factor analytic models: unidimensional, 5-factor correlated, second-order and bi-factor model, and contrasts them with the results of Yuan et al. (2019) and Ramsenthaler et al. (2022) for the 5-factor model. In addition, the cutoff values are given using the dynamic model fit index. The unidimensional model has the worst fit (CFI = 0.452; RMSEA = 0.217). The second-order model has worse AIC and BIC values compared to the 5-factor and bi-factor models and is only just above the limit for CFI (0.932) and RMSEA (0.078). The bi-factor model achieves good values with a CFI of 0.956, RMSEA of 0.067 and low AIC and BIC. However, the 5-factor model already has an acceptable fit when the thresholds (OP method equivalent to Hu and Bentler (1999) and also DDM method) are taken into account: CFI of 0.949 (OP: > 0.929; DDM > 0.933), RMSEA of 0.070 with 90% CI of 0.065, 0.074 (OP: < 0.089; DDM < 0.081), SRMR of 0.058 (OP: < 0.067).

The comparison of the baseline models shows that the 5-factor model has an acceptable fit and supports the concept of Yuan et al. (2019). Therefore, the 5-factor model was tested in depth.

Table 2 shows the standardized factor loadings, as well as the alpha and omega values of each factor. The loadings range from 0.67 to 0.87, reaching an acceptable level. Alpha ranges from 0.807 to 0.875 and Omega from 0.811 to 0.875, indicating good reliability.

**Table 2:** Confirmatory factor analysis for 5-factor model (total sample).

Factor - help	Standardized factor loading	Alpha/Omega
DRS1 - I feel like I should do more for the world.	0.73	0.854/0.858
DRS2 - I feel a strong urge to help other people.	0.85	
DRS3 - I want to be a more generous person.	0.87	
Factor - live		
DRS4 - I make plans for my life.	0.76	0.839/0.840
DRS5 - I reflect on the things I still want to do.	0.84	
DRS6 - I am motivated to try new things.	0.80	
Factor - perspective		
DRS7 - I can let go of the little problems.	0.70	0.807/0.811
DRS8 - I am able to stop sweating the small stuff.	0.79	
DRS9 - I am less stressed about the things that are bothering me.	0.81	
Factor - legacy		
DRS10 - I think about what legacy I will have left behind.	0.67	0.837/0.845
DRS11 - I reflect on whether people will think of me after death.	0.87	
DRS12 - I reflect on how I will be remembered.	0.87	
Factor - connection		
DRS13 - I want to spend more time with the people I care about.	0.84	0.875/0.875
DRS14 - I want to tell the people I care about how I feel about them.	0.87	
DRS15 - I want to spend more time with my family.	0.79	

Results from multi-group equality testing are presented in Table 3. In the first step, it was tested whether the CFA model fit in each group is acceptable. For this purpose, specific cutoff values were calculated based on the DFI approach (DDM; estimator: maximum likelihood; cutoff precision: 3-decimal point; misspecification level: fair = mean absolute discrepancy 0.05).

**Table 3:** Measurement invariance for 5-factor model.

Model	X^2^	df	CFI	RMSEA [90% CI]	Δ*X*^2^	Δdf	*p*	AIC
50–59	411.98	80	0.942	0.078 [0.071, 0.086]	-	-	-	491.98
DFI (50–59)^1^	-	-	> 0.936	< 0.083	-	-	-	-
60–69	342.46	80	0.929	0.080 [0.072, 0.089]	-	-	-	422.46
DFI (60–69)^1^	-	-	> 0.924	< 0.086	-	-	-	-
70+	298.43	80	0.952	0.066 [0.058, 0.074]	-	-	-	378.43
DFI (70+)^1^	-	-	> 0.935	< 0.079	-	-	-	-
Configural invariance (baseline)	1052.9	240	0.942	0.043 [0.041, 0.046]	-	-	-	-
Metric	1075.1	260	0.942	0.042 [0.039, 0.044]	22.2	20	0.330	-
Scalar	1111.8	280	0.941	0.041 [0.038, 0.043]	36.7	20	0.013	-
Partial scalar invariance	1090.4	276	0.942	0.040 [0.038, 0.043]	21.4	16	0.164	-
Strict invariance	1153.1	306	0.939	0.039 [0.037, 0.042]	62.7	30	0.000	-

1 Dynamic Fit Index - DDM method calculated with Wolf and McNeish (2024).

df, degrees of freedom; CFI, comparative fit index; RMSEA, Root mean square error of approximation; CI, confidence interval; AIC, Akaike’s information criterion.

Models have an acceptable fit in all three age groups and achieve the best fit in the group of 70 years and older (CFI = 0.952; RMSEA = 0.066), thus meeting requirements for testing measurement invariance. Metric invariance across age groups was reached (*Δ*X^2^ = 22.2; Δdf = 20; *p* = 0.330). For scalar invariance, chi-square test (Δ*X*^2^ = 36.7; Δdf = 20; *p* = 0.013) indicated that there is no full scalar invariance. Therefore, the source of non-invariance was investigated by sequentially releasing item intercept constraints. By freeing intercepts of DRS1 – ‘I feel I should do more for the world’ (factor: motivation to help) and DRS4 – ‘I make plans for my life’ (factor: motivation to live) partial scalar invariance was obtained (Δ*X*^2^ = 21.4; Δdf = 16; *p* = 0.164). In the final step residual (strict) invariance was tested showing a significant worsening in overall model fit (Δ*X*^2^ = 62.7; Δdf = 30; *p* = 0.000), which indicates that at least one item residual is different across age groups. In summary, the measurement invariance is acceptable, as further explained in the discussion, and allows for mean comparison of the latent factors. The descriptive results of the DRS for the three chronical age groups and the five factors are presented in Table 4. In addition, the scores for the total sample and the total score are given to better illustrate the results.

**Table 4:** Scale descriptives in chronical age groups.

	Total sample	Chronical age groups			
		50–59	60–69	70+	*p^3^*
Total M (SD)^1^	39.76 (12.42)	40.16 (13.37)	39.98 (11.94)	39.14 (11.72)	0.289
Skewness (SE)	−0.26 (0.06)	−0.22 (0.09)	−0.25 (0.11)	−0.35 (0.10)	
Kurtosis (SE)	0.54 (0.12)	0.34 (0.19)	0.40 (0.21)	0.87 (0.20)	
*Alpha/Omega*	0.864/0.865	0.877/0.877	0.854/0.855	0.856/0.857	
Motivation to help M (SD)^2^	5.44 (3.76)	5.41 (4.00)	5.45 (3.71)	5.45 (3.52)	0.972
Skewness (SE)	0.13 (0.06)	0.22 (0.09)	0.12 (0.11)	0.00 (0.1)	
Kurtosis (SE)	−0.80 (0.12)	−0.82 (0.19)	−0.83 (0.21)	−0.81 (0.20)	
*Alpha/Omega*	0.854/0.858	0.871/0.874	0.832/0.837	0.853/0.858	
Motivation to live M (SD)^2^	8.76 (3.80)	9.10 (3.91)_a_	8.81 (3.75)_**a,b**_	8.36 (3.68)_**b**_	0.002
Skewness (SE)	−0.53 (0.06)	−0.62 (0.09)	−0.51 (0.11)	−0.51 (0.10)	
Kurtosis (SE)	−0.24 (0.12)	−0.18 (0.19)	−0.21 (0.21)	−0.28 (0.20)	
*Alpha/Omega*	0.839/0.840	0.848/0.848	0.831/0.831	0.834/0.835	
Putting life into perspective M (SD)^2^	9.48 (3.43)	9.31 (3.72)	9.65 (3.27)	9.50 (3.23)	0.250
Skewness (SE)	−0.59 (0.06)	−0.56 (0.09)	−0.55 (0.11)	−0.61 (0.10)	
Kurtosis (SE)	0.25 (0.12)	−0.04 (0.19)	0.40 (0.22)	0.42 (0.20)	
*Alpha/Omega*	0.807/0.811	0.826/0.828	0.785/0.790	0.800/0.806	
Legacy M (SD)^2^	4.75 (4.02)	4.86 (4.24)	4.51 (3.89)	4.81 (3.88)	0.291
Skewness (SE)	0.58 (0.06)	0.59 (0.09)	0.61 (0.11)	0.51 (0.10)	
Kurtosis (SE)	−0.54 (0.12)	−0.66 (0.19)	−0.45 (0.21)	−0.51 (0.20)	
*Alpha/Omega*	0.837/0.845	0.855/0.861	0.810/0825	0.835/0.842	
Connection to others M (SD)^2^	11.34 (3.43)	11.48 (3.54)_**a**_	11.56 (3.32)_**a**_	11.01 (3.37)_**b**_	0.010
Skewness (SE)	−1.04 (0.06)	−1.09 (0.09)	−1.06 (0.11)	−1.01 (0.10)	
Kurtosis (SE)	0.88 (0.12)	0.85 (0.19)	0.88 (0.21)	0.99 (0.20)	
*Alpha/Omega*	0.875/0.875	0.878/0.879	0.876/0.878	0.868/0.868	
*n*	1806	670	524	612	

Subscript letters indicate significant differences based on the post-hoc test.^1^Range: 0–75; ^2^Range: 0–15, ^3^ANOVA (Welch’s Test; *Post-hoc*: Games-Howell).

M, mean; SD, Standard Deviation; SE, Standard Error.

The total score for the entire sample is just over half the scale (*M* = 39.76, SD = 12.42); ranked by score, the order of the factors is: legacy (*M* = 4.75, SD = 4.02), motivation to help (*M* = 5.44, SD = 3.75), motivation to live (*M* = 8.76, SD = 3.80), putting life into perspective (*M* = 9.48, SD = 3.43), connection to others (*M* = 11.34, SD = 3.43). Cronbach’s alpha (0.785–0.878) and McDonald’s omega (0.790–0.789) were calculated for all groups and indicate acceptable to good reliability. The values of skewness and kurtosis indicate also acceptable to good symmetrical and normal distribution. The mean values seem relatively stable across the chronical age groups, with only a slight, but significant decrease for the two factors ‘motivation to live’ (*p* = 0.002) and ‘connection to others’ (*p* = 0.010). The post-hoc test shows – see subscript letters in Table 4 – that the group of people aged 70 and over differs significantly (live: *M* = 8.36; connection *M* = 11.01), while the other three dimensions are not related to chronological age.

The same procedure was used for the perceived age variable. Table 5 shows the descriptive results of the DRS for the three perceived age groups, the five factors and additionally for the total score. Cronbach’s alpha (0.779 to 0.883) and McDonald’s omega (0.795 to 0.883) indicate acceptable to good reliability, and values of skewness and kurtosis indicate also acceptable to good symmetrical and normal distribution. In addition to a significant decrease in the total score for people who consider themselves older than their chronological age (*M* = 36.05, *p* < 0.001), this phenomenon can also be observed in the two factors ‘motivation to live’ (*M* = 7.05, *p* < 0.001) and ‘putting life into perspective’ (*M* = 8.11, *p* < 0.001). Although not significant, the same trend can also be observed for the factor ‘connection to others’, while the other two dimensions are not correlated with perceived age.

**Table 5:** Scale descriptives in perceived age groups.

	Total sample	Preceived age groups			
		younger	same	older	*p^3^*
Total M (SD)^1^	39.76 (12.42)	40.14 (12.33)_**a**_	39.73 (12.99)_**a**_	36.05 (12.16)_**b**_	< 0.001
Skewness (SE)	−0.26 (0.06)	−0.27 (0.06)	−0.15 (0.19)	−0.30 (0.20)	
Kurtosis (SE)	0.54 (0.12)	0.61 (0.13)	0.572 (0.37)	−0.05 (0.39)	
*Alpha/Omega*	0.864/0.865	0.865/0.867	0.871/0.874	0.837/0.830	
Motivation to help M (SD)^2^	5.44 (3.76)	5.43 (3.74)	5.59 (3.92)	5.27 (3.76)	0.749
Skewness (SE)	0.13 (0.06)	0.11 (0.6)	0.17 (0.19)	0.32 (0.20)	
Kurtosis (SE)	−0.80 (0.12)	−0.85 (0.13)	−0.66 (0.37)	−0.49 (0.39)	
*Alpha/Omega*	0.854/0.858	0.855/0.859	0.846/0.848	0.858/0.861	
Motivation to live M (SD)^2^	8.76 (3.80)	8.97 (3.77)_a_	8.53 (3.82)_a_	7.05 (3.60)_**b**_	< 0.001
Skewness (SE)	−0.53 (0.06)	−0.58 (0.06)	−0.51 (0.19)	−0.33 (0.20)	
Kurtosis (SE)	−0.24 (0.12)	−0.15 (0.13)	−0.32 (0.37)	−0.57 (0.39)	
*Alpha/Omega*	0.839/0.840	0.843/0.843	0.845/0.846	0.779/0.795	
Putting life into perspective M (SD)^2^	9.48 (3.43)	9.60 (3.37)_**a**_	9.60 (3.34)_**a**_	8.11 (3.80)_**b**_	< 0.001
Skewness (SE)	−0.59 (0.06)	−0.61 (0.06)	−0.68 (0.19)	−0.19 (0.20)	
Kurtosis (SE)	0.25 (0.12)	0.32 (0.13)	0.78 (0.37)	−0.38 (0.39)	
*Alpha/Omega*	0.807/0.811	0.799/0.803	0.815/0.817	0.842/0.857	
Legacy M (SD)^2^	4.75 (4.02)	4.72 (3.98)	4.91 (4.34)	4.78 (4.10)	0.839
Skewness (SE)	0.58 (0.06)	0.59 (0.06)	0.52 (0.19)	0.54 (0.20)	
Kurtosis (SE)	−0.54 (0.12)	−0.50 (0.12)	−0.74 (0.37)	−0.71 (0.39)	
*Alpha/Omega*	0.837/0.845	0838/0.846	0.860/0.869	0.797/0.811	
Connection to others *M* (SD)^2^	11.34 (3.43)	11.42 (3.38)	11.11 (3.48)	10.83 (3.72)	0.88
Skewness (SE)	−1.04 (0.06)	−1.07 (0.06)	−0.99 (0.19)	−0.86 (0.20)	
Kurtosis (SE)	0.88 (0.12)	1.00 (0.12)	0.77 (0.37)	0.13 (0.39)	
*Alpha/Omega*	0.875/0.875	0.883/0.883	0.827/0.829	0.856/0.856	
*n*	1806	1,481	173	152	

Subscript letters indicate significant differences based on the post-hoc test.^1^Range: 0–75; ^2^Range: 0–15, ^3^ANOVA (Welch’s Test; *Post-hoc*: Games-Howell).

M, mean; SD, Standard Deviation; SE, Standard Error.

George and Mallery (2021, p. 115f): Skewness: 0 = symmetrical distribution (cut off: excellent −1/+1; acceptable − 2/+2); Kurtosis (‘excess’ kurtosis): 0 = normal distribution (cut off: excellent −1/+1; −2/+2 acceptable).

Table 6 shows the correlation results of the DRS with the Big Five dimensions. It is noteworthy that almost all dimensions of the two instruments correlate with each other. Exceptions are conscientiousness and legacy (*p* = 0.611) and neuroticism and connection to others (*p* = 0.341). The directions of the effects in the neuroticism dimension contribute to a stabilization of the total score (*p* = 0.459), demonstrating the importance of considering the DRS as a 5-factor model. The strongest significant correlations are found between extraversion and ‘motivation to live’ (*r*_p_ = 0.215) and between neuroticism and ‘putting life in perspective’ (*r*_p_ = −0.228).

**Table 6:** Correlations with death reflection scale and Big Five.

Variable	Big Five									
	Extraversion		Agreeableness		Conscientiousness		Neuroticism		Openness	
	*r_p_*	*p*	*r_p_*	*p*	*r_p_*	*p*	*r_p_*	*p*	*r_p_*	*p*
DRS total	0.201	< 0.001	0.141	< 0.001	0.125	< 0.001	0.017	0.459	0.151	< 0.001
Motivation to help	0.059	0.012	**0.139**	< 0.001	0.058	0.013	0.091	< 0.001	0.086	< 0.001
Motivation to live	**0.215**	< 0.001	0.072	0.002	**0.153**	< 0.001	−0.035	0.133	**0.173**	< 0.001
Putting life into perspective	**0.134**	< 0.001	0.079	< 0.001	**0.104**	< 0.001	**−0.228**	< 0.001	**0.109**	< 0.001
Legacy	0.078	< 0.001	0.055	0.019	−0.012	0.611	**0.178**	< 0.001	0.069	0.003
Connection to others	**0.199**	< 0.001	**0.136**	< 0.001	**0.131**	< 0.001	0.022	0.341	0.071	0.002

Statistical test: *r* (Pearson); values marked in bold: *r*_p_ > 0.1.

## Discussion

The aim of this study was to test the reliability and validity of the Death Reflection Scale in a population sample of older people. A comparison of the baseline model shows that the bi-factor model appears to have the best fit, considering the magnitude of the individual scores, except for the SRMR, confirming the results of Ramsenthaler et al. (2022). However, the bi-factor model also requires higher DFI cutoff values to more accurately reflect misfit quantification (Murray and Johnson, 2013). On the other hand, the 5-factor model also shows a good fit. Given this variability, Morgan et al. (2015) argue that models must also be judged on substantive and conceptual grounds. Conceptually, the DRS was developed by Yuan et al. (2019) as a 5-factor model. The authors do not mention a general latent factor itself; accordingly, the specific factors should be interpreted as subscales. The bi-factor model would indicate a general factor as representative of something like general positive processing of death, while the specific factors would be representative of a common aspect of concrete positive behavioral intentions within each dimension that is not captured by the general factor. For example, research on cognitive abilities has benefited from bi-factorial models (e.g., Betts et al., 2011). However, the present analysis shows that the items load low on the g-factor (8 items below 0.5; highest value 0.61), while at the same time relatively high loadings can be found on the specific factors (12 items above 0.5; highest value 0.89), indicating a systematic deviation from the variance explained by the general factor (Dunn and McCray, 2020). When loadings on the specific factors are high, DeMars (2013) argues that score estimates for the specific factors can be meaningful as long as score users are aware that the subscales reflect information beyond the general score. On the other hand, bi-factor models can lead to serious identification and estimation problems when predicting criteria in multiple regression frameworks (Eid et al., 2018). Overall, the five-factor model appears to be the better solution and has been further tested.

The CFAs of the 5-factor model for the entire sample as well as for subsamples show good values and support its applicability: factor loadings are at a good level, the same applies to alpha and omega – only in the age group 60–69 the values in the dimension of ‘putting life into perspective’ fall below 0.8 at 0.785 and 0.790, but are still acceptable. To assess if the instrument is interpreted in the same way across age groups, measurement invariance was tested. The configural factorial measurement invariance results suggest that older respondents of different ages use an identical cognitive framework when processing death reflection – these results are in line with Ramsenthaler et al. (2022). Also metric invariance was achieved across age groups, which indicates that items contribute to the five latent factors to a similar degree.

Full scalar invariance was not achieved – following Putnick and Bornstein (2016), the source of non-invariance was investigated by sequentially releasing item intercept constraints. Partial scalar invariance was obtained by freeing the intercepts of DRS1 – ‘I feel I should do more for the world’ (factor: motivation to help) and DRS4 - ‘I make plans for my life’ (factor: motivation to live), indicating non-identical scale properties for these two items across age groups. However, cross-group comparisons of subscale scores are supported when at least two indicators per construct are invariant (Steenkamp and Baumgartner, 1998), which is the case for all five subscales of the DRS. Since this key requirement is met, mean comparisons can be made between groups of older people. However, whether comparisons across the entire lifespan are permissible should be examined in more detail. Ramsenthaler et al. (2022) show with their sample that even full metric invariance across age groups – < 30, 31–40, 41–50, and > 50 – was not reached and assume that mortality cues have different salience across the lifespan (for different responses across age groups see, e.g., Maxfield et al., 2014, 2017; Roberts and Maxfield, 2019).

Testing the construct validity showed only a partial confirmation of proposed relationships, similar to Ramsenthaler et al. (2022). For chronological age, only H1b – the decrease in ‘motivation to live’ – can be confirmed. Although not significant, there is a slight increase in the dimension of ‘putting life into perspective’ (H1c), which corresponds to the assumption. Contrary to the hypothesis, there is a significant decrease in the dimension of ‘connection to others’ (H1e). It should be noted that it is not the quantity of the relationship that becomes more important in old age, but rather, in the context of socioemotional selectivity (Carstensen, 1992; Carstensen et al., 1999), its quality (Lansford et al., 1998). However, the wording of items DRS10 and DRS12 (‘spend more time’) is more quantitatively oriented, which may explain a decrease in the oldest old. In the context of perceived age, hypotheses H2b (decrease in ‘motivation to live’) and H2c (decrease in “putting life into perspective”) can be confirmed. In addition, the data point, although not significantly, in the direction of H2e, while ‘motivation to help’ and ‘legacy’ are not related to perceived age. Two assumptions can be made: It is known that subjective age is a good marker for poor health (Voegeli et al., 2021), which is related to possibility (Abolfathi Momtaz et al., 2014) and thus to introspective evaluation regarding helping others. The stability of ‘motivation to help’ could be an expression of contradictory processes (desire versus evaluation of the actual possibility). The stability in the area of ‘legacy’ can be explained on the basis of Waggoner et al. (2023) that other drivers, in addition to the anxiety of death, may be responsible for the pursuit of a legacy, such as the extension of one’s self-narratives and the maintenance of one’s narrative identity. Narrative identity is characterized by stability and change over the life course (Reischer, 2021) and is therefore likely to be accompanied by recurrent reflections on one’s legacy. The stability of the DRS score may be an expression of this ongoing process.

In the final part of this paper, DRS was exploratively correlated with the Big Five personality traits. It is striking that almost all dimensions show significant correlations, with 12 showing considerable strength. Taking into account the personality traits and their facets (Costa and McCrae, 1992), the convergent validity with the BFI-10 was confirmed by Rammstedt et al. (2017). Therefore, the found correlations become explicable. For the personality trait Extraversion, the facets gregariousness, assertiveness, and positive emotions should explain the positive correlations with ‘motivation to live’ (Rott and Jopp, 2006), ‘putting life in perspective’ (Sharma, 2011), and ‘connection to others’ (Rapp et al., 2019). With regard to Agreeableness, it is altruism and willingness for cooperation that provide an explanation for the correlation between ‘motivation to help’ (Graziano et al., 2007) and ‘connection to others’ (McCrae and Costa, 2006). In the domain of Conscientiousness, the facets of dutifulness and deliberation in the sense of self-controlled composure may explain the ‘motivation to live’, the capacity to form ‘connections with others’ (Rapp et al., 2019), and the ability to put ‘life into perspective’ (Melendez et al., 2019). Impulsiveness, anxiety and vulnerability should explain the negative correlation with Neuroticism and the DRS dimensions ‘putting life into perspective’ (Chochinov et al., 2006) and positive correlation with ‘legacy’. Openness is positively related to life satisfaction (Stephan, 2009), which in turn is particularly strongly related to ‘motivation to live’(Bornet et al., 2021). A study by Cox et al. (2010) also showed that generativity, which is also reflected in the DRS, and the personality traits Extraversion and Openness are significantly related, which is also consistent with the present study.

Due to the digital divide and the particularly limited number of frail older people using the Internet, the limitations of online surveys need to be considered. In this context, even if people were selected from a big online panel, there are likely to be limitations in the representativeness of the sample. In addition, the strength of the study in testing the DRS with a large number of older people reaches its limits when it comes to testing validity, as the assumptions of Yuan et al. (2019) relate to the entire life course. Therefore, the insignificant correlations in the sample of older people could reflect attitudes that become more entrenched with age, or interactions of factors that seem to stabilize the values of the DRS. In any case, further research on the DRS, especially with other psychometric instruments, seems useful.

In conclusion, the DRS shows acceptable quality in the present study, and its application to older people appears possible. An example of how it can be used is the correlation of the DRS with the Big Five, which shows the relationships between personality traits and prosocial attitudes in the context of death awareness. Further work with the DRS – recommended as a 5-factor model – in gerontological and psycho-gerontological research seems worthwhile to better understand how older people process positive aspects of death and their behavioral intentions to realize such positive aspects, or how the DRS develops over the life course and what (social) life events influence the DRS. In addition, it would be useful to look more closely at the links with concepts such as successful and active aging or generativity (Villar et al., 2023) – for example, correlations with the Loyola Generativity Scale (McAdams et al., 1992). The particular value of the DRS lies in the fact that it views the awareness of death not as a threat, but as a potential. From a gerontopsychological point of view, this insight is of central importance and is reflected in the generativity of older people. It would be good to foster this perspective of death awareness and perhaps remove some of the taboos surrounding death.

## Data Availability

The data used in this study are available through the authors upon reasonable request.
